# Evaluation of Muscle Oxygenation Responses to Eccentric Exercise and Recovery Enhancement Using Capacitive–Resistive Electric Transfer and Vibration Therapy

**DOI:** 10.3390/jcm15020794

**Published:** 2026-01-19

**Authors:** Łukasz Oleksy, Anna Mika, Maciej Daszkiewicz, Martyna Sopa, Miłosz Szczudło, Maciej Kuchciak, Artur Stolarczyk, Olga Adamska, Paweł Reichert, Zofia Dzięcioł-Anikiej, Renata Kielnar

**Affiliations:** 1 Department of Orthopedics, Traumatology and Hand Surgery, Faculty of Medicine, Wroclaw Medical University, 50-556 Wroclaw, Poland; loleksy@oleksy-fizjoterapia.pl (Ł.O.); pawel.reichert@umw.edu.pl (P.R.); 2 Institute of Physical Culture Sciences, University of Szczecin, 71-065 Szczecin, Poland; 3 Oleksy Medical & Sport Sciences, 37-100 Łańcut, Poland; 4 Institute of Clinical Rehabilitation, University of Physical Culture in Kraków, 31-571 Kraków, Poland; 5 Physiotherapy Research Laboratory, University Centre of Physiotherapy and Rehabilitation, Faculty of Physiotherapy, Wroclaw Medical University, 50-368 Wroclaw, Poland; maciej.daszkiewicz@student.umw.edu.pl; 6 Institute of Applied Mechanics, Faculty of Mechanical Engineering, Poznan University of Technology, 61-138 Poznań, Poland; martyna.sopa@put.poznan.pl; 7 Faculty of Physical Culture Sciences, Collegium Medicum, University of Rzeszów, 35-959 Rzeszów, Poland; mszczudlo@ur.edu.pl (M.S.); mkuchciak@ur.edu.pl (M.K.); 8 Department of Orthopaedics and Rehabilitation, Medical and Dentistry Faculty, Medical University of Warsaw, 02-091 Warsaw, Poland; artur.stolarczyk@wum.edu.pl; 9 Department of Ophthalmology, Faculty of Medicine, Collegium Medicum Cardinal Stefan Wyszyński University in Warsaw, 01-815 Warsaw, Poland; o.adamska@uksw.edu.pl; 10 Department of Rehabilitation, Medical University of Bialystok, 24A M. Skłodowskiej-Curie St., 15-276 Bialystok, Poland; zofia.dzieciol-anikiej@umb.edu.pl; 11 Institute of Physiotherapy, University Center for Research and Development in Health Sciences, Faculty of Health Sciences and Psychology, Collegium Medicum, University of Rzeszów, 35-315 Rzeszów, Poland; kielnarrenata@o2.pl

**Keywords:** TECAR, vibration therapy, eccentric exercise, muscle fatigue, muscle saturation, NIRS, sport injury, recovery

## Abstract

**Background:** Although Capacitive–Resistive Electric Transfer (TECAR) and vibration therapy (VT) are increasingly used in sports recovery, their effects on muscle oxygenation remain unclear. **Objectives:** This study compared the short-term influence of TECAR and VT on muscle oxygenation following eccentric exercise in young, active adults. We hypothesized that both interventions would support early metabolic recovery, as reflected by changes in muscle oxygenation, and potentially reduce the risk of musculoskeletal overuse. **Methods:** Forty-one young, recreationally active adults (age: 19 ± 2 years; height: 168 ± 9 cm; body mass: 63 ± 13 kg) were randomized into two groups: TECAR therapy and VT. Muscle oxygenation was assessed at baseline, post-exercise, and post-intervention using the arterial occlusion method with a MOXY muscle oxygenation monitor (Fortiori Design LLC, USA). The primary variables were mVO_2_ (muscle oxygen consumption), ΔSmO_2_ (change in oxygen saturation during occlusion), and ΔtHb (change in hemoglobin level during occlusion). Data were analyzed using a two-way repeated-measures ANOVA with post hoc Tukey tests, and statistical significance was set at *p* < 0.05. **Results:** Eccentric exercise significantly reduced mVO_2_ in both groups (VT: −0.18 ± 0.40 to −1.62 ± 0.70; TECAR: −0.12 ± 0.40 to −1.24 ± 0.70), indicating decreased metabolic demand. Following recovery, mVO_2_ increased in both groups (VT: −0.86 ± 0.50; TECAR: −0.35 ± 0.40), with no significant between-group differences (*p* > 0.05). ΔSmO_2_ also decreased after exercise (VT: −0.7 ± 0.4 to −3.2 ± 0.9; TECAR: −0.9 ± 0.6 to −3.45 ± 0.7). After recovery, ΔSmO_2_ partially returned to baseline (VT: −2.6 ± 0.8; TECAR: −1.35 ± 0.4), with no significant between-group differences. ΔtHb increased following exercise in both groups (VT: 0.03 ± 0.04 to 0.13 ± 0.09; TECAR: 0.03 ± 0.04 to 0.15 ± 0.07) and decreased after recovery to similar levels (VT: −0.05 ± 0.05; TECAR: −0.06 ± 0.04; *p* > 0.05). **Conclusions:** Both TECAR and VT were associated with improved muscle oxygenation during early recovery after eccentric exercise, as reflected by increases in mVO_2_ and comparable ΔtHb responses. Although ΔSmO_2_ tended to decrease more after VT, this difference was not statistically significant and should be interpreted cautiously. Overall, both modalities appear to be effective recovery-supporting strategies, while further controlled studies are needed to clarify their role in different athletic populations and exercise contexts.

## 1. Introduction

Eccentric muscle contractions, frequently encountered in athletic disciplines involving braking, landing, or rapid changes of direction, are among the main triggers of exercise-induced muscle damage (EIMD) [1,2,3]. These movements impose substantial mechanical strain on myofibrils, causing microstructural disruptions that initiate local inflammation and modify afferent neural signaling [3,4]. One of the best-recognized manifestations of EIMD is delayed-onset muscle soreness (DOMS), typically developing within the first 24 h after exercise and characterized by movement-related pain, muscle stiffness, loss of maximal voluntary strength, limited joint mobility, and disturbed proprioceptive control [3,5]. Eccentric contractions impose high mechanical strain on sarcomeres, leading to microstructural disruptions such as Z-line streaming and myofibrillar damage [1,3]. These alterations trigger secondary events, including calcium dysregulation, inflammatory responses, and transient impairments in muscle microcirculation [2,3,4,5]. The inflammatory cascade involves cytokines such as IL-6 and TNF-α and sensitization of group III and IV afferent fibers, contributing to pain perception and altered neuromuscular control [3,4,5]. DOMS is therefore associated with soreness, strength loss, and reduced metabolic efficiency. Efficient recovery strategies are critical for maintaining performance and reducing injury risk, especially in high-intensity or high-frequency training environments. While a range of conventional recovery methods—such as stretching, cryotherapy, massage, hydrotherapy, or compression—are commonly applied, their physiological efficacy remains inconclusive, with research showing variable outcomes [6,7,8,9]. Targeted modalities such as TECAR and VT have gained attention. However, direct physiological comparisons using objective metabolic markers are limited.

Among emerging recovery modalities, capacitive–resistive electric transfer (TECAR) and vibration therapy (VT) have gained increasing attention due to their capacity to stimulate deep tissue structures and support metabolic and vascular processes involved in post-exercise recovery [10,11]. TECAR therapy employs high-frequency electromagnetic currents (typically 300 kHz–1 MHz) to induce controlled endogenous tissue heating, which promotes vasodilation, nitric oxide–mediated endothelial responses, enhanced perfusion, and improved metabolic exchange, thereby facilitating the clearance of metabolic byproducts and supporting muscle relaxation and regeneration [10,12,13]. In contrast, vibration therapy applies localized mechanical oscillations that activate muscle spindles and Ia afferents, increasing neuromuscular excitability and enhancing microvascular perfusion through a muscle-pump mechanism, which may accelerate oxygen resaturation and the removal of metabolites such as lactate and hydrogen ions [14,15,16,17]. Both interventions have been shown to attenuate neuromuscular fatigue, improve contraction efficiency, and reduce the severity of DOMS [1,15,18].

Despite their increasing use in sports medicine and rehabilitation, the physiological mechanisms underlying the recovery effects of TECAR and VT remain insufficiently characterized. In particular, the assessment of muscle oxygenation dynamics—especially within deeper tissue layers—offers an objective and sensitive indicator of local recovery processes [19,20]. Traditional measures of fatigue and recovery, such as perceived soreness or performance tests, provide limited insight into the microvascular and metabolic adaptations that occur within the muscle following eccentric stress. In contrast, near-infrared spectroscopy (NIRS)-based evaluation of deep muscle oxygen saturation (SmO_2_) enables continuous, non-invasive monitoring of tissue oxygen extraction and hemodynamic recovery patterns, reflecting the balance between oxygen delivery and utilization [20,21,22]. TECAR therapy and vibration therapy are widely used in contemporary sports medicine and physiotherapy practice as recovery-supporting interventions. However, despite their popularity, direct physiological comparisons between these modalities—particularly with respect to muscle oxygenation—remain limited. By applying NIRS to compare TECAR and VT following eccentric exercise, the present study advances understanding of short-term metabolic recovery responses. A comparative evaluation may therefore provide clinically relevant insight into their distinct recovery-related mechanisms.

A large body of research shows that NIRS is a reliable and physiologically meaningful method for assessing muscle oxygenation and post-exercise recovery [23,24,25]. Southern et al. [22] reported good test–retest reproducibility for NIRS-derived measures of muscle oxygen consumption and mitochondrial capacity, with coefficients of variation of 10–11% for the mVO_2_ recovery constant across different exercise modalities. Because this parameter reflects mitochondrial oxidative capacity, it is sensitive to even subtle physiological changes after fatiguing exercise. NIRS also provides data for blood oxygenation, blood flow, and oxidative function in a single non-invasive assessment [20,22,23,24,26]. It is important to note that changes in muscle oxygen saturation do not directly represent metabolic recovery but rather reflect the dynamic balance between oxygen delivery, oxygen extraction, and mitochondrial oxidative capacity. While increased oxygen availability may facilitate recovery processes, true metabolic restoration depends on mitochondrial function and the efficiency of oxidative phosphorylation. Therefore, NIRS-derived parameters should be interpreted as indirect markers of metabolic status rather than definitive indicators of recovery. In the present study, particular emphasis was placed on context-dependent changes in ΔSmO_2_ during vascular occlusion as an indicator of functional oxidative capacity rather than resting metabolic demand. These features make NIRS particularly well suited to studying the multidimensional recovery process after eccentric muscle damage and justify its use as the primary tool for monitoring fatigue and regeneration in the present study.

Given that both TECAR and VT are proposed to modulate blood flow and metabolic exchange within muscle tissue, analyzing deep muscle oxygenation profiles may provide critical insight into their actual physiological effectiveness. Therefore, the present study aimed to compare the short-term effects of TECAR therapy and vibration therapy on muscle oxygenation and perfusion-related responses following eccentric exercise in young, recreationally active adults. We hypothesized that both modalities would be associated with favorable changes in post-exercise muscle oxygenation during early recovery. This study should be considered a preliminary investigation conducted in young, recreationally active adults, intended to establish baseline physiological responses prior to future studies in elite athletes, older individuals, and clinical populations.

## 2. Materials and Methods

This study was designed as a randomized, short-term experimental trial and was conducted in a controlled laboratory setting, where all exercise and recovery interventions were performed.

### 2.1. Study Participants

A total of forty-one healthy, physically active adults volunteered to participate in the study (Table 1). Participants were randomly assigned to one of two intervention groups:

Group 1 (n = 20)—VT recovery

Group 2 (n = 21)—TECAR recovery

**Table 1:** Subjects’ characteristics.

	Group 1	Group 2
Number of subjects (n)	20	21
Male/Female	3/17	4/17
Height (cm)	168 ± 10	167 ± 7
Weight (kg)	63 ± 14	64 ± 12
Age	19 ± 2	19 ± 2

**Figure 1:**
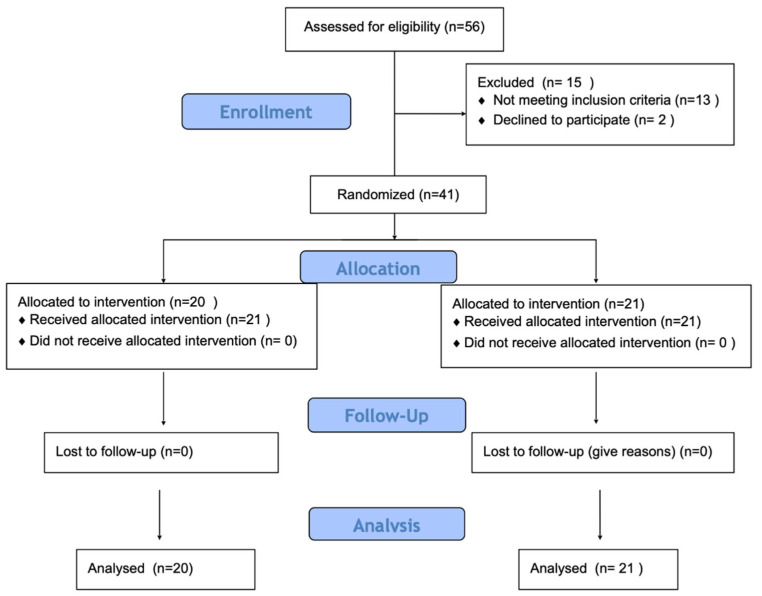
Study flow diagram.

Inclusion criteria included age between 18 and 25 years, regular engagement in physical activity (minimum three sessions per week), absence of lower-limb pain, injury, or surgery during the preceding 12 months, and no cardiovascular or neurological disorders. Recruitment was conducted via online university announcements and student social media networks. Participants were assigned to intervention groups based on order of enrollment, a quasi-randomization approach frequently used in preliminary physiological research. While this method does not ensure full allocation concealment, it allows for balanced group sizes and feasibility in exploratory study designs (Figure 1).

Prior to participation, each subject received a detailed explanation of the study aims and procedures and provided written informed consent. All experimental procedures conformed to the ethical principles of the 1964 Declaration of Helsinki and its subsequent amendments. Ethical approval was obtained from the Ethical Committee of the Regional Medical Chamber in Kraków (35/KBL/OIL/2024).

### 2.2. Study Design

Testing was conducted by the same research team to ensure procedural consistency. Participants’ body height (cm) and mass (kg) were recorded at baseline. A standardized 10-min warm-up consisting of dynamic lower-limb movements preceded the fatigue protocol.

**Figure 2:**
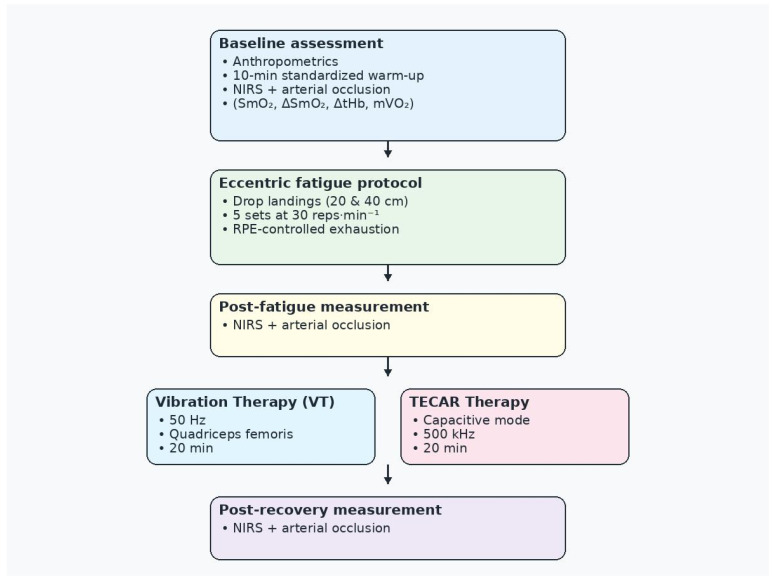
Study design and experimental timeline. NIRS—near-infrared spectroscopy; RPE—Rating of Perceived Exertion scale; TECAR—capacitive–resistive electric transfer therapy; VT—vibration therapy.

Only the right leg was evaluated. Measurements were performed at three time points: pre-exercise baseline, immediately after the eccentric fatigue protocol, and after the recovery intervention (Figure 2).

### 2.3. Procedures

#### 2.3.1. Muscle Oxygenation Measurement

Muscle oxygen saturation (SmO_2_) and total hemoglobin (tHb) were assessed using near-infrared spectroscopy (NIRS) with the MOXY Muscle Oxygen Monitor (Fortiori Design LLC, Hutchinson, MN, USA). Measurements were performed on the right vastus lateralis (VLO) muscle.

##### Measurement Setup

Participants were positioned in a semi-recumbent posture (60° hip flexion) with knees fully extended. A pneumatic cuff for arterial occlusion was placed proximally around the thigh, immediately inferior to the inguinal fold, to enable transient blood flow restriction. Importantly, cuff placement was spatially separated from the NIRS measurement site. The MOXY sensor was securely attached to the skin over the belly of the vastus lateralis muscle, approximately midway between the greater trochanter and the lateral femoral condyle, using medical adhesive and covered with an optically opaque sleeve to minimize ambient light interference. To minimize inter-individual variability related to adipose tissue thickness, the NIRS sensor was placed on the most palpable region of the muscle belly, where muscle tissue was directly accessible beneath the skin. All sensor placements were performed by the same experienced investigator.

##### Arterial Occlusion Protocol

The arterial occlusion method was employed to assess muscle oxygen kinetics. The cuff was inflated to a pressure of 250–275 mmHg, adjusted individually to ensure complete arterial occlusion, and maintained for 30 s, followed by a 30 s reperfusion phase. Complete occlusion was confirmed by the rapid decline in SmO_2_ immediately after cuff inflation and the absence of a pulsatile signal during the occlusion phase. Two occlusion–reperfusion cycles were completed, separated by a 30 s rest interval [21,22]. Data were continuously collected through the VO_2_ Master application and subsequently exported for analysis in MATLAB 25.2 (MathWorks, Natick, MA, USA). The mean values derived from the two occlusion cycles were used for statistical analysis.

##### Variables

**ΔSmO**_2_** (Change in Oxygen Saturation During Occlusion)**—represents the drop in muscle oxygen saturation that occurs during an occlusion period and serves as an indicator of the muscle’s oxygen utilization. Expressed as a relative value in % SmO_2_, this parameter reflects how efficiently a muscle extracts and consumes oxygen when blood flow is temporarily restricted. A larger, more negative ΔSmO_2_ denotes faster oxygen extraction during vascular occlusion and reflects the functional oxidative capacity of the muscle. Depending on the physiological context, this may indicate either increased metabolic demand (e.g., post-exercise fatigue) or preserved/enhanced oxidative function following recovery. Conversely, a smaller ΔSmO_2_ drop may reflect reduced oxygen utilization due to either metabolic normalization at rest or diminished functional capacity, depending on the physiological condition of the muscle. In post-recovery assessments, a more negative ΔSmO_2_ value indicates that the recovery intervention enhances muscle regeneration, whereas minimal change suggests ineffective recovery or persistent fatigue.

**ΔtHb (Change in Hemoglobin Level During Occlusion)**—reflects changes in muscle blood volume during an occlusion period and provides insight into vascular reactivity and microcirculatory function. A positive ΔtHb value indicates blood pooling beneath the cuff, which is associated with good vascular responsiveness, effective muscle perfusion, healthy microcirculation, and enhanced recovery capacity. In contrast, a negative ΔtHb value indicates displacement of blood from the muscle, reduced perfusion, inadequate vascular response, and potentially muscle overload or insufficient oxygen supply.

**mVO**_2_** (Muscle Oxygen Consumption)**—calculated from the rate of SmO_2_ decline during arterial occlusion and reflects the metabolic activity and oxidative capacity of the muscle. Derived from the relationship between changes in total hemoglobin (tHb) and muscle oxygen saturation (SmO_2_), mVO_2_ quantifies how quickly a muscle consumes oxygen when blood inflow is temporarily restricted. Expressed in μM-HbDiff/s, higher mVO_2_ values signify greater oxygen utilization, better metabolic conditioning, more efficient mitochondrial function, and faster post-exercise recovery. Conversely, lower mVO_2_ values indicate fatigue, impaired mitochondrial function, reduced blood flow, or an absent regenerative response.

#### 2.3.2. Fatiguing Eccentric Protocol

To induce controlled eccentric fatigue, participants performed drop landings from two consecutive platforms (20 cm and 40 cm heights) at a frequency of 30 repetitions per minute. The protocol consisted of five sets of landings, separated by 1 min rest intervals, following a method described in previous studies [27]. This procedure effectively elicited eccentric overload in the quadriceps femoris muscle, promoting measurable muscle fatigue. To further standardize the level of fatigue across participants, perceived exertion was monitored using the Rating of Perceived Exertion (RPE) scale, and the protocol was continued until a comparable level of voluntary exhaustion was reached.

#### 2.3.3. VT Intervention

The VT recovery intervention was applied using a Vibra 3.0 pneumatic vibration device (Vibra Plus, A Circle S.p.A., San Pietro in Casale, Bologna, Italy). Localized mechanical vibrations were delivered at a frequency of 50 Hz to the quadriceps femoris using a 5 cm cup-shaped transducer for a duration of 20 min. The vibration amplitude and pneumatic pressure were set according to the manufacturer’s standardized preset corresponding to moderate stimulation and were kept constant across all participants. The device operated within a range of 10–300 Hz, and the chosen frequency corresponded to the range commonly associated with increased blood flow and neuromuscular activation. All VT sessions were administered by the same trained operator following an identical application protocol to ensure intervention fidelity.

#### 2.3.4. TECAR Intervention

The TECAR intervention was administered using a BACK4 device (Winback Europa, Biarritz, France) operating in capacitive (CAP) mode at a frequency of 500 kHz and an intensity of 40% (up to 300 W maximum output). This relative intensity setting corresponds to a moderate therapeutic dose commonly used in clinical and experimental applications and was selected to ensure participant comfort and safety. The electrode was applied to the quadriceps femoris with continuous movement in a distal-to-proximal direction using standardized moderate manual pressure. Each treatment lasted 20 min. During the procedure, participants were seated, and a metallic return electrode was positioned beneath the thigh to complete the electrical circuit. All TECAR interventions were delivered by the same experienced therapist using an identical protocol to minimize operator-dependent variability.

#### 2.3.5. Data Analysis

In this study, the levels of tHb and SmO_2_ were measured over time. Each test lasted approximately 2.5 min, during which 30 s phases of occlusion and relaxation alternated. For each occlusion and relaxation phase, the following variables were calculated [21,28,29,30]:

- Change in hemoglobin level during occlusion (ΔtHb): defined as the difference between the average tHb value in the last five seconds and the first five seconds of the occlusion phase:

$$\Delta tHb={tHb}_{end}-{tHb}_{start}$$

where tHb_end_ is the average tHb level in the last five seconds of the test and tHb_start_ is the tHb level in the first five seconds of the test.

- Change in saturation level (ΔSmO_2_): defined as the difference between the average SmO_2_ value in the last five seconds and the first five seconds of the occlusion phase:

$$\Delta {SmO}_{2}={{SmO}_{2}}_{end}-{{SmO}_{2}}_{start}$$

where SmO_2end_ is the average SmO_2_ level in the last five seconds of the test and SmO_2start_ is the SmO_2_ level in the first five seconds of the test.

- Muscle oxygen consumption rate (mVO_2_): calculated based on changes in tHb and SmO_2_ values during the test, using the following relationship:

$$SmO_{2}\left(t\right)=\frac{O_{2}Hb\left(t\right)}{tHb\left(t\right)}$$

After rearrangement:

$$O_{2}Hb\left(t\right)=SmO_{2}\left(t\right)\cdot tHb\left(t\right)$$

Applying the product rule for derivatives:

$$\frac{dO_{2}Hb}{dt}=tHb\left(t\right)\frac{dSmO_{2}}{dt}+SmO_{2}\frac{dtHb}{dt}$$

Assuming that during occlusion, oxygen supply is blocked, the decrease in oxyhemoglobin is related to local oxygen consumption. Therefore,

$$m\overset{˙}{V}O_{2}\approx -\left({\overset{¯}{tHb}}_{start}\frac{\Delta SmO_{2}}{\Delta t}+{\overset{¯}{SmO_{2}}}_{start}\frac{\Delta tHb}{\Delta t}\right)$$

where mVO_2_ is the muscle oxygen consumption rate (μM-Hbdiff/s), ${\overset{¯}{tHb}}_{start}$ is the average tHb value before occlusion, and ${\overset{¯}{SmO_{2}}}_{start}$ is the average SmO_2_ value before occlusion. The remaining factors are the changes (slopes) in SmO_2_ and tHb values during occlusion.

### 2.4. Statistical Analysis

The normality of data distribution was verified with the Shapiro–Wilk test, confirming that the analyzed variables met the assumptions for parametric testing. A two-way repeated-measures ANOVA (group × time) was used to assess differences in muscle oxygenation indices between the TECAR and VT groups across three measurement time points (baseline, post-fatigue, and post-recovery). Post hoc pairwise comparisons were conducted using the Tukey test when significant interactions were identified. Statistical significance was set at *p* < 0.05. Effect sizes (Cohen’s d) were calculated to estimate the magnitude of observed differences and were interpreted as follows: small (0.2–0.3), medium (0.5), and large (>0.8) [31]. A priori power analysis indicated that a minimum of 30 participants was required to achieve a statistical power of 0.80 at an alpha level of 0.05, assuming an expected effect size of d = 0.8 and an intraclass correlation coefficient (ICC) of at least 0.50. The power analysis was based on expected within-subject changes in muscle oxygenation parameters across time points rather than on between-group differences, given the repeated-measures design and the acute nature of the interventions. All statistical analyses were performed using the STATISTICA 13.0 software package (TIBCO Software Inc., Palo Alto, CA, USA).

## 3. Results

### 3.1. Changes in ΔSmO_2_ (Change in Oxygen Saturation During Occlusion)

**Figure 3:**
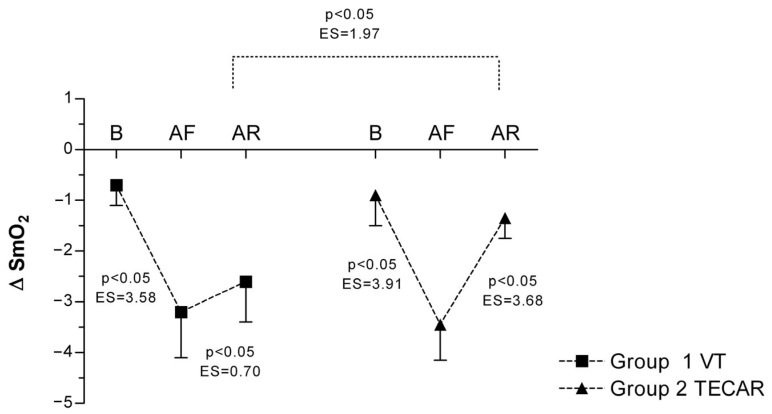
Differences in ΔSmO_2_ between groups and across three measurements. Values are expressed as mean ± SD; *p*—*p*-value; ES—effect size; ΔSmO_2_ (Change in Oxygen Saturation During Occlusion); B (baseline); AF (after fatigue); AR (after recovery).

Muscle oxygen saturation during occlusion (ΔSmO_2_) showed a pronounced decrease after eccentric exercise in both groups, consistent with increased oxygen extraction. In the VT group, ΔSmO_2_ decreased from −0.7 ± 0.4 at baseline to −3.2 ± 0.9 post-fatigue and remained at −2.6 ± 0.8 after recovery. In the TECAR group, ΔSmO_2_ declined from −0.9 ± 0.6 at baseline to −3.45 ± 0.7 post-fatigue and recovered to −1.35 ± 0.4 post-intervention. Although ΔSmO_2_ values tended to remain more negative in the VT group after recovery, no statistically significant between-group differences were observed (Figure 3).

### 3.2. Changes in ΔtHb (Change in Hemoglobin Level During Occlusion)

**Figure 4:**
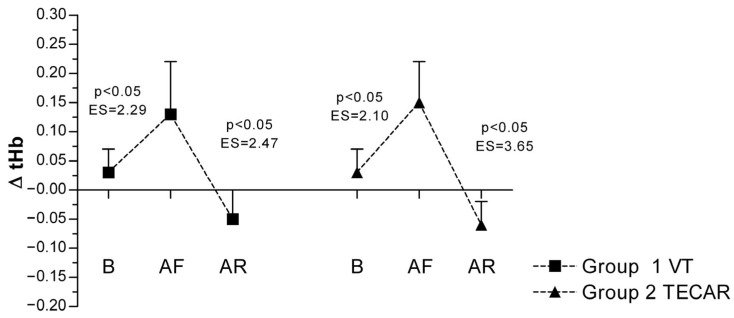
Differences in ΔtHb between groups and across three measurements. Values are expressed as mean ± SD; *p*—*p*-value; ES—effect size; ΔtHb (Change in Hemoglobin Level During Occlusion); B (baseline); AF (after fatigue); AR (after recovery).

In response to eccentric exercise, an increase in ΔtHb was observed in both groups, reflecting exercise-induced changes in local blood volume. In the VT group, ΔtHb increased from 0.03 ± 0.04 at baseline to 0.13 ± 0.09 post-fatigue and decreased to −0.05 ± 0.05 following recovery. Comparable responses were observed in the TECAR group (baseline: 0.03 ± 0.04; post-fatigue: 0.15 ± 0.07; post-recovery: −0.06 ± 0.04). No significant between-group differences were found at any time point, indicating similar perfusion-related responses to both recovery modalities (Figure 4).

### 3.3. Changes in mVO_2_ (Muscle Oxygen Consumption)

**Figure 5:**
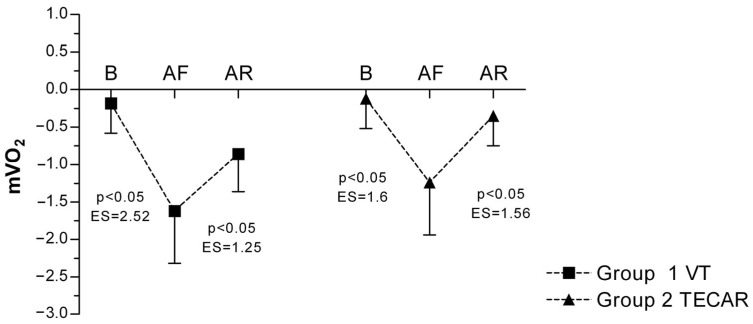
Differences in mVO_2_ between groups and across three measurements. Values are expressed as mean ± SD; *p*—*p*-value; ES—effect size; mVO_2_ (Muscle Oxygen Consumption); B (baseline); AF (after fatigue); AR (after recovery).

In both groups, a significant decrease in mVO_2_ was observed following eccentric exercise compared with baseline, indicating increased metabolic demand. In the VT group, mVO_2_ decreased from −0.18 ± 0.40 at baseline to −1.62 ± 0.70 post-fatigue, and partially recovered to −0.86 ± 0.50 after the recovery intervention. Similarly, in the TECAR group, mVO_2_ declined from −0.12 ± 0.40 at baseline to −1.24 ± 0.70 post-fatigue and increased to −0.35 ± 0.40 post-recovery. A significant main effect of time was observed, whereas no significant group or group × time interaction effects were detected, indicating comparable recovery-related changes in both interventions (Figure 5).

## 4. Discussion

The purpose of this study was to examine the short-term responses of selected muscle oxygenation and perfusion-related parameters following two commonly used recovery-supporting modalities, TECAR therapy and vibration therapy, after eccentric exercise in young, recreationally active adults. The analysis revealed clear physiological changes in muscle oxygenation following exercise and during the subsequent recovery period. Both groups demonstrated a marked reduction in mVO_2_ after exercise, consistent with an acute increase in metabolic demand and exercise-induced fatigue. Following recovery, mVO_2_ increased significantly in both the VT and TECAR groups, indicating a partial restoration of oxidative metabolism. Importantly, no significant between-group differences were observed in post-recovery mVO_2_ values, suggesting that both interventions were associated with comparable short-term effects on this parameter. Similarly, ΔtHb responses did not differ significantly between groups, indicating a broadly similar influence of both recovery modalities on muscle perfusion. While ΔSmO_2_ tended to remain more negative following recovery in the VT group, this difference did not reach statistical significance and should therefore be interpreted cautiously. Overall, both recovery interventions were associated with favorable changes in muscle oxygenation-related parameters, without clear evidence of superiority of one modality over the other. This observation corresponds with earlier studies demonstrating that TECAR and vibratory stimulation can alleviate symptoms of post-exercise muscle fatigue and enhance motor performance [10,11,15]. The present results also reinforce previous evidence emphasizing that selecting appropriate recovery strategies plays a crucial role in restoring muscle function after physical activity [7,8,15]. Traditional recovery approaches, including massage, compression garments, or cryotherapy, have produced mixed and often inconsistent effects [7,8,9]. Therefore, interest has shifted toward more targeted methods—such as radiofrequency-based thermotherapy and vibration interventions—which, as indicated by the current study, may provide effective support for post-exercise muscle recovery.

The observed improvements in oxygenation-related parameters are indicative of short-term physiological responses associated with both TECAR and vibration therapy rather than definitive mechanistic effects. TECAR, through the application of high-frequency electromagnetic currents, has been proposed to induce deep endogenous heating that may promote vasodilation and microcirculation [10,12,13]. Such responses could be associated with the post-recovery increases in mVO_2_; however, the absence of significant between-group differences suggests that both recovery modalities were linked to comparable effects on this parameter. Similarly, the lack of significant differences in ΔtHb between groups indicates that both TECAR and vibration therapy elicited broadly similar perfusion-related responses, consistent with previous reports describing generalized vascular effects of these interventions. Vibration therapy has been associated with mechanical and neuromuscular stimulation that may influence local circulation and oxygen resaturation [14,15,16,17]; however, the present findings do not allow firm conclusions regarding modality-specific perfusion mechanisms. Although ΔSmO_2_ tended to remain more negative following recovery in the VT group, this between-group difference did not reach statistical significance and should therefore be interpreted cautiously. A more negative ΔSmO_2_ reflects a higher rate of oxygen extraction during occlusion, which may indicate differences in oxidative response patterns rather than superior recovery outcomes. In line with previous studies reporting beneficial effects of vibration therapy on post-exercise muscle function [1,15,18], the current results suggest that both TECAR and vibration therapy are associated with favorable short-term changes in muscle oxygenation, while differences between modalities remain modest and require confirmation in controlled studies.

However, the existing literature presents mixed evidence regarding vibration-induced changes in muscle oxygenation and recovery [1,16,17,32,33]. Some studies report clear improvements in muscle blood flow: Manimmanakorn et al. [17] showed that post-exercise whole-body vibration increased the tissue oxygenation index, oxygenated hemoglobin, and total hemoglobin, and reduced deoxygenated hemoglobin compared with both active and passive recovery. Other reports demonstrated limited effects of vibration treatment on metabolic or performance variables and highly variable analgesic outcomes [1,15,18,31]. Other investigations similarly describe inconsistent effects on peripheral circulation, venous return, muscle oxygenation, and total hemoglobin [17,34,35,36]. Proposed mechanisms include vibration-induced increases in regional hemoglobin saturation, enhanced venous return via a muscle-pump mechanism, and tonic vibration reflex–driven rises in metabolic demand and vasodilation [1,2,16,17,35,36,37,38]. Stronger mechanistic support comes from Percival et al. [2], who found that local VT (45 Hz) accelerated SmO_2_ resaturation and improved strength retention after eccentric exercise, likely through improved blood flow, modulation of vascular tone, and attenuation of inflammation-related microvascular dysfunction. In the context of these findings, our results contribute new evidence by showing that both VT and TECAR therapy effectively modulate muscle oxygenation during recovery, reflected by favorable changes in mVO_2_ and ΔtHb. Importantly, only ΔSmO_2_ indicated a trend toward a different metabolic response after VT; however, this observation did not reach statistical significance and should not be interpreted as evidence of greater effectiveness.

Extensive research indicates that eccentric exercise–induced muscle damage (EIMD) disrupts both skeletal muscle function and the local regulation of microcirculation, leading to impaired oxygen delivery and utilization [1,2,3,36,37]. These disturbances manifest as reduced tissue oxygen saturation and an altered balance between oxygen supply and metabolic demand, making NIRS-derived variables particularly sensitive markers of fatigue and declining performance [34,36]. Importantly, NIRS can also effectively assess the restoration of tissue oxygenation during recovery, a finding supported by the present results. The observed changes in saturation parameters (mVO_2_, ΔSmO_2_, ΔtHb) following TECAR and VT demonstrate that both modalities meaningfully support the re-establishment of muscle metabolic function after exercise, while simultaneously highlighting the utility of NIRS as a tool for monitoring the effectiveness of different recovery interventions.

Evidence from NIRS research shows that hemoglobin saturation adjusts rapidly to exercise intensity and that moderate eccentric muscle damage does not necessarily disrupt oxygen extraction [34,37,39,40]. It was suggested that when eccentric exercise is low or moderate in intensity, oxygen supply appears to recover rapidly, meaning that unchanged saturation in such settings reflects maintained microvascular function rather than an absence of physiological disturbance [40]. In our study, recreationally active participants were exposed to a relatively demanding eccentric task. This likely contributed to the pronounced post-exercise reductions in saturation that we observed, particularly in mVO_2_ and ΔSmO_2_, as less-trained individuals typically exhibit larger saturation fluctuations due to lower oxidative efficiency. Indeed, research shows that well-trained individuals can function effectively at lower levels of oxygenation and demonstrate smaller relative changes in saturation, while recreationally trained subjects experience greater variability [34,40]. Similar training-status effects have been documented in cycling and drop-set resistance protocols, where trained individuals show deeper deoxygenation without impaired performance [24,38,39]. Therefore, muscle oxygenation cannot be interpreted as a universal indicator of fatigue without considering training level, habitual movement patterns, and sport-specific physiology.

The inconsistent findings across studies examining post-exercise changes in muscle saturation following vibration or TECAR therapy may stem from factors unrelated to the intrinsic efficacy of these methods. The presence or absence of saturation changes does not necessarily reflect their regenerative effectiveness—especially since numerous studies report neuromuscular, mechanical, or perceptual benefits of vibration that occur independently of oxygenation metrics. Although NIRS is a sensitive tool, saturation outcomes are influenced by local perfusion capacity, individual microvascular architecture, adipose tissue thickness, and muscle recruitment patterns during both exercise and recovery [35,40,41]. These sources of variability likely contribute to the divergent results reported in the literature. Despite these complexities, our findings indicate that both TECAR and vibration therapy meaningfully modulate post-exercise oxygenation, as reflected in improvements in mVO_2_ and ΔtHb. Notably, only ΔSmO_2_ showed a trend toward a different pattern of oxidative activity following recovery, warranting further investigation.

Evidence for the effectiveness of both VT and TECAR in supporting recovery after eccentric exercise is reinforced by our previous work, where both modalities improved neuromuscular function [42]. This is consistent with reports showing that electromyographic changes parallel the dynamics of muscle oxygenation: Yamada et al. [41] demonstrated strong correlations between reductions in electromyography mean power frequency and changes in concentrations of oxygenated and deoxygenated hemoglobin and myoglobin during sustained muscle contractions, which were driven by intracellular disturbances such as decreased pH and the accumulation of potassium ions. In our study, mVO_2_, ΔSmO_2_, and ΔtHb showed similar fatigue–recovery patterns after eccentric exercise and following VIBRA 3.0 or TECAR, supporting the sensitivity of NIRS as an indicator of neuromuscular recovery. From a practical perspective, the present findings indicate that both TECAR and vibration therapy may be considered viable short-term recovery-supporting options following eccentric exercise. In the absence of clear between-group differences, the selection of one modality over the other should be based on individual tolerance, logistical availability, and the specific recovery context rather than expectations of superior physiological effects.

It should be highlighted that several methodological factors also make NIRS data difficult to interpret. Reoxygenation behaves differently during active versus passive recovery, and adipose tissue thickness can greatly reduce NIRS sensitivity [19,22,35]. In addition, without using arterial occlusion, NIRS cannot distinguish whether changes reflect altered blood flow or altered oxygen consumption [19]. Resting mVO_2_ and blood flow also show considerable natural variability [22,41]. Therefore, the lack of changes in muscle oxygenation after recovery interventions in some studies may be due to factors such as low metabolic stress, the specific muscle measured, timing issues, or methodological limitations rather than a true absence of physiological effects. Taken together, NIRS is a valuable tool for monitoring fatigue and recovery, but interpreting its results requires considering muscle characteristics, training status, exercise intensity, and technical constraints.

This study has several limitations that should be acknowledged. First, the relatively small sample size and the restriction to young, recreationally active adults limit the generalizability of the findings. Second, the analysis focused exclusively on immediate post-intervention responses, and no functional or performance-based outcomes were included. These measures are part of a larger research project and will be reported separately. Although near-infrared spectroscopy provides valuable insight into muscle oxygenation dynamics, parameters such as mVO_2_, ΔSmO_2_, and ΔtHb are sensitive to methodological factors, including adipose tissue thickness, probe placement, and individual differences in microvascular structure, which may have influenced the precision of the recorded responses. In addition, the absence of direct physiological markers (e.g., blood lactate concentration or inflammatory indicators) limits the ability to draw mechanistic conclusions regarding the pathways through which TECAR and vibration therapy may influence muscle metabolism and perfusion. Future research should incorporate larger cohorts, controlled study designs, longer follow-up periods, and a broader range of exercise and recovery protocols to better characterize recovery dynamics. Investigations involving competitive or professional athlete populations and the inclusion of functional performance outcomes would further clarify the practical relevance of TECAR and vibration therapy in high-performance and injury-prevention contexts.

## 5. Conclusions

This study provides a direct comparison of TECAR and vibration therapy with respect to short-term changes in muscle oxygenation following eccentric exercise. Both interventions were associated with significant post-recovery increases in mVO_2_, indicating improved oxygen utilization during the recovery period in young, physically active individuals. Perfusion-related responses assessed through ΔtHb did not differ between groups, indicating that both modalities exerted a comparable influence on muscle blood volume during occlusion. Although ΔSmO_2_ values tended to show a more pronounced post-recovery decrease in the vibration therapy group, this between-group difference did not reach statistical significance and should therefore be interpreted with caution. Rather than indicating superior recovery, this finding may reflect differences in oxygen extraction dynamics during occlusion within the limits of the present study design. Overall, the findings support the use of both TECAR and VT as viable recovery-supporting modalities associated with favorable short-term changes in muscle oxygenation parameters following eccentric exercise, without clear evidence of superiority of one intervention over the other. While vibration therapy may elicit distinct oxygenation response patterns, the present data do not allow definitive conclusions regarding enhanced oxidative re-engagement. TECAR likewise demonstrated beneficial effects, particularly in relation to perfusion-related parameters, which were comparable between interventions. Future studies employing controlled designs, larger sample sizes, longer follow-up periods, and functional or performance-based outcomes are warranted to clarify the physiological relevance and practical implications of saturation-related responses in post-exercise recovery.

## Data Availability

All data generated or analyzed during this study are included in this published article.
